# *Eichhornia crassipes* Ameliorated Rheumatoid Arthritis by Modulating Inflammatory Cytokines and Metalloproteinase Enzymes in a Rat Model

**DOI:** 10.3390/medicina59091594

**Published:** 2023-09-04

**Authors:** Sara Sattar, Arham Shabbir, Muhammad Shahzad, Tasleem Akhtar, Arfan Ahmad, Sulaiman Mohammed Alnasser, Bushra Riaz, Shaik Karimullah, Ashfaq Ahmad

**Affiliations:** 1 Department of Pharmacology, Faculty of Pharmacy, The University of Lahore, Defence Road Campus, Lahore 54000, Pakistan; dr.sarasattar@imchospital.com.pk; 2 Department of Pharmacology, Institute of Pharmacy, Faculty of Pharmaceutical and Allied Health Sciences, Lahore College for Women University, Jail-Road, Lahore 54000, Pakistan; 3 Department of Pharmacology, University of Health Sciences, Lahore 54000, Pakistan; shahzad912@uhs.edu.pk (M.S.); tasleem.akhtar@uhs.edu.pk (T.A.); 4 University Diagnostic Laboratory, University of Veterinary and Animal Sciences, Lahore 54000, Pakistan; iffivet@uvas.edu.pk; 5 Department of Pharmacology and Toxicology, Unaizah College of Pharmacy, Qassim University, Buraydah 52571, Saudi Arabia; sm.alnasser@qu.edu.sa; 6 Department of Pharmacy Practice, College of Pharmacy, University of Hafr Al Batin, Hafar Al Batin 39524, Saudi Arabia; bushrariaz@uhb.edu.sa (B.R.); kshaik@uhb.edu.sa (S.K.); ashfaqa@uhb.edu.sa (A.A.)

**Keywords:** inflammation, cytokines, rheumatism, medicinal plants, water hyacinth

## Abstract

*Background and Objectives*: This study was planned to investigate the anti-arthritic property of flowers of *E. crassipes* in a Sprague–Dawley rat model by administering Freund’s Complete Adjuvant (FCA). *Materials and Methods*: Arthritis was induced at day 0 in all rats except negative controls, while arthritic progress and paw edema were analyzed on specific days (8th, 13th, 18th, and 23rd) via the macroscopic arthritic scale and a digital Vernier caliper, respectively. Histopathological parameters were examined using a Hematoxylin and Eosin (H&E) staining method. Blood samples were withdrawn from rats to investigate the effects of the *E. crassipes* flower on the mRNA expression values of inflammatory markers, via a reverse transcription PCR technique. Serum samples were used to determine prostaglandin E2 (PGE2) levels using enzyme-linked immunosorbent assay (ELISA). Values of alanine transaminase (ALT), aspartate aminotransferase (AST), creatinine, and urea, besides hematological parameters, i.e., the hemoglobin (Hb) content and complete blood count (CBC), were investigated. *Results*: The data showed that *E. crassipes* inhibited the arthritic progress and ameliorated the paw edema. The amelioration of parameters assessed via the histopathological analysis of ankle joints, as well as via hematological analysis, confirmed the diminution of rheumatoid arthritis (RA) in the plant-treated groups. Treatment with *E. crassipes* inhibited the expression levels of tumor necrosis factor-α (TNF-α), interleukins (IL-1β and IL-6), nuclear factor KappaB (NF-κB), matrix metalloproteinase (MMP-2 and MMP-3), and vascular endothelial growth factor (VEGF). Serum PGE2 levels were also found to be reduced in treatment groups. A biochemical investigation revealed the improvements in hepatic markers in plant-treated groups. The data indicated that the plant has no hepatotoxic or nephrotoxic effects at the studied dose. GC-MS (Gas Chromatography-Mass Spectrometry) analysis displayed the presence of phytochemicals having known anti-inflammatory and antioxidant properties. *Conclusions*: Therefore, it may be concluded that *E. crassipes* possesses anti-arthritic characteristics that could be attributed to the modulation of pro-inflammatory cytokines, MMPs, and PGE2 levels.

## 1. Introduction

Rheumatoid Arthritis (RA) is an autoimmune disorder of connective tissues, especially the joints, cartilage, and bones, causing their disability and other systemic disorders. Its effects involve synovial inflammation, proliferation, hyperplasia, and the distortion of cartilage and bone [1]. The World Health Organization (WHO) confirmed that arthritis is the 31st primary reason for YLD (years lived with disability) globally [2]. The risk of RA is predominant in women as compared to in men. Moreover, it increases with the passage of time [3]. RA starts with the invasion of the synovial membrane in the cartilage. In typical RA, the synovial membrane turns into inflammatory tissue, “Pannus”, which destroys surrounding cartilage and bone [4]. The borderline between the pannus and cartilage is occupied by activated macrophages and synovial fibroblasts that produce matrix metalloproteinases [5]. The extra-articular manifestation may also develop as a result of sub-standard pharmacotherapeutic management [6].

The pathogenesis of RA involves both immune cells and inflammatory mediators of our defense system [4]. It includes cellular proliferation in the synovial layer and the infiltration of pro-inflammatory cytokines, hormones, and chemotactic and growth factors. This results in local pannus formation, and thus, the destruction of cartilage, bone, and soft tissues occur [7].

Non-steroidal anti-inflammatory drugs (NSAIDs) are the first-line therapeutic agents for RA, such as, piroxicam, ibuprofen, aceclofenac, and naproxen. They minimize the pain, stiffness of joints, and inflammation by down-regulating pro-inflammatory cytokines, like prostaglandins and thromboxane, but cannot eliminate the root cause. They also cause gastrointestinal disturbances, asthma, and renal toxicity [8]. Short-term opioids have proven their efficacy for RA, such as fentanyl and oxycodone, especially in pain management. But the long-term ones are linked to reduced efficacy and side effects, like respiratory depression, dependence, and tolerance [9]. Disease-modifying anti-rheumatic drugs (DMARDs) are a novel drug strategy towards RA, such as, methotrexate, sulfasalazine, hydroxychloroquine, azathioprine, cyclosporine, and leflunomide [10]. They perform by modifying different processes of immunomodulatory and inflammatory responses that are responsible for the clinical manifestations of RA [11]. But the use of DMARDs comes with an increased risk of tuberculosis infection. Methotrexate and leflunomide may result in side-effects, like skin allergy, gastrointestinal disturbances, diarrhea, and abnormalities in liver enzymes. Azathioprine can cause nausea, vomiting, abdominal pain, and diarrhea, while cyclosporine affects the renal functions [10]. TNF inhibitors have been declared as the target specific treatment for RA, by blocking TNF and decreasing the inflammatory response [12], but they also have an increased risk of tuberculosis [13]. Targeting immunological components for RA therapy is being considered for those who do not respond to methotrexate or DMARDs, such as T-cell inhibition but the available biological therapies are very costly [14].

*Eichhornia crassipes* (Mart.) Solms is a free-floating vascular aquatic plant, belonging to family Pontederiaceae [15]. Traditionally, the plant has been used to treat inflammatory conditions. Preliminary pharmacological studies also highlighted the plant’s anti-inflammatory property [16]. A previously conducted phytochemical analysis of *E. crassipes* also showed various phytochemicals possessing anti-inflammatory and antioxidant characteristics [17]. 

To the best of our knowledge, data regarding the anti-arthritic potential of *E. crassipes* is scarce; therefore, this study was planned to evaluate the anti-arthritic effect of *E. crassipes*, using FCA-induced arthritis in a Sprague–Dawley rat model.

## 2. Materials and Methods

### 2.1. Plant Collection and Identification

*Eichhornia crassipes* was gathered from the natural pond near Jallo Park (3–6 km), Lahore (10 km east), in Punjab Province, during summers (June). The plant was authenticated by the Department of Botany, Government College University, Lahore (Voucher # GC. Herb. Bot. 3640).

### 2.2. Preparation of Methanol Extract and Fractions

The collected flowers of the plant were washed, air-dried under the shade, and powdered using an electrical grinder. Then, maceration was performed by soaking ground powder in methanol, using an air-tight glass jar. The jar was stored at room temperature, with recurrent shaking, for 7 days. The methanolic extract was passed through a Whatman filter and subsequently thickened in a rotary evaporator. 

The crude methanol extract (50 g) was mixed in distilled water (500 mL). This mixture was then mixed with *n*-hexane (100 mL) for liquid–liquid extraction, using a separating funnel. The *n*-hexane fraction was separated, and its solvent was vaporized under reduced pressure, via a rotary evaporator [18]. After that, the aqueous layer was taken to fractionation with ethyl acetate (100 mL), using the same method [19]. The percentage yield of the methanol extract, *n*-hexane fraction, and ethyl acetate fraction were calculated and were found to be 47.64%, 2.14%, and 1.105%, respectively. 

### 2.3. Test Animals

Sprague–Dawley rats of both sexes, with a weight range of 150–250 g, were subjected to an investigation of the anti-arthritic property of the plant. Sprague–Dawley rats were procured from the University of Veterinary & Animal Sciences, Lahore. Rats were administered a standard pellet diet and water *ad libitum*. They were kept under 12 h dark/light cycles, and average conditions of humidity (60–70%) and temperature of (28 °C ± 2 °C) were continued during this study tenure. They were familiarized to their surroundings for the duration of one week earlier than the trial [20].

### 2.4. Assessment of Anti-Arthritic Effect

Thirty-six Sprague–Dawley rats were dispersed into 6 groups. Except in the negative control (group 1), arthritis was induced in the rest of the 5 groups by injecting FCA (0.1 mL) into the sub-plantar area, starting from day 0. Group 1 and the arthritic control (group 2) were fed with normal saline (NS) orally [21], from day 8 to 22. The reference group (group 3) was dosed with piroxicam (10 mg/kg b.w., i.p.) [22]. Group 4 was given methanol extract, group 5 was given the *n*-hexane fraction, and group 6 was dosed with the ethyl acetate fraction (100 mg/kg b.w., p.o., each), from day 8 to 22 [16].

### 2.5. Measurement of Arthritic Score and Paw Volume

Morphological features of the arthritis were analyzed macroscopically for all the rats on the 8th, 13th, 18th, and 23rd day [21]. A score of 0 was used for a normal paw. Scores of 1 to 2 were for mild-to-moderate swelling and erythema of the digits. A score of 3 was given to severe swelling and erythema of the digits. A score of 4 was given to a significant deformity and incapability of the movement of limbs. The paw volume was also recorded on the same days using a digital Vernier caliper.

### 2.6. Histopathological Evaluation

The ankle joints of all groups, injected with FCA, were separated and fixed with 10% formalin and afterwards decalcified with a decalcifying agent (10% formic acid with 10% formalin). Then, samples were fixed with paraffin and stained using an H&E method, after cutting them into slices of a 5 µm thickness. Arthritic parameters, such as bone eruption, the formation of a pannus, and the intrusion of inflammatory cells, were analyzed. Results were predicted based on a scale having a range of 0, 1, 2, 3, and 4 for normal, mild, moderate, and gross variations, respectively [23].

### 2.7. Analysis of mRNA Expression Values of the Pro-Inflammatory Cytokines TNF-α, IL-1β, IL-6, NF-κB, MMP-2, MMP-3, and VEGF

Blood samples of rats were collected, and RNA was extracted using the Total RNA Isolation (TRIzol) method. A Nanodrop spectrophotometer (major science mini-300) was used to quantify the total RNA extracted. Then, cDNA was generated by following the RevertAid First Strand cDNA synthesis kit’s protocol (Thermo Scientific, Waltham, MA, USA, LOT 00960732). GAPDH was taken as a house-keeping gene. Primers for IL-1β, MMP-2, and MMP-3 were formulated manually. Sequences of other primers, i.e., IL-6, TNF-α, NF-κB, and VEGF, were used from previously published papers, as exhibited in Table 1. cDNA (1 µL) was centrifuged with the forward–reverse primer mix (1 µL), nuclease-free water, and PCR Master Mix (5 µL). The thermal cycler was operated for 35 cycles of 3 phases: denaturation (95 °C for 10 s), annealing (according to temperatures displayed in Table 1 for 20 s), and subsequently, extension (72 °C for 30 s) cycles.

### 2.8. Determination of PGE2 Levels

The Rat Prostaglandin E2 ELISA kit (Bioassay Technology Laboratory, Shangai, China, Cat No. E-0504Ra) was used to determine PGE2 levels. Results were obtained by measuring the Optical Density (OD) using an ELISA reader (BIORAD, Hercules, CA, USA, PR 4100) at a 450 nm wavelength.

### 2.9. Assessment of Hematological Parameters

A commercial automated hemocytometer was selected to calculate the hemoglobin (Hb) content, as well as white blood cells (WBCs), red blood cells (RBCs), and platelet (PLT) counts. 

### 2.10. Biochemical Parameters

Commercial kits processed the serum samples to calculate the values of AST, ALT, urea, and creatinine, using an automated chemistry analyzer.

### 2.11. GC-MS Investigation

The *E. crassipes* methanol extract was analyzed using an Agilent 5975 MSD (USA) with a 7890B GC, with a DB-5MS column (30 m, 0.25 mm, 0.25 mm). The GC injector temperature was maintained at 230 °C. The oven temperature was 70 °C for 1 min, then increased up to 320 °C at 10 °C/min, and then maintained at 320 °C for 1 min. The transfer line temperature was 250 °C. Helium was used as the carrier gas. The carrier gas flow rate was maintained at 1 mL/min. The MS source was operated in electron impact mode at 70 eV. The MS was scanned from 40 to 500 m/z.

### 2.12. Statistical Analysis

Data were stated as the mean ± SD, using one-way analysis of variance (ANOVA), then post hoc Tukey’s test, where applicable, to arbitrate the significant difference among groups. Here, *p* < 0.05 was reflected as significant.

## 3. Results

### 3.1. E. crassipes Repressed Arthritic Development

**Figure 1:**
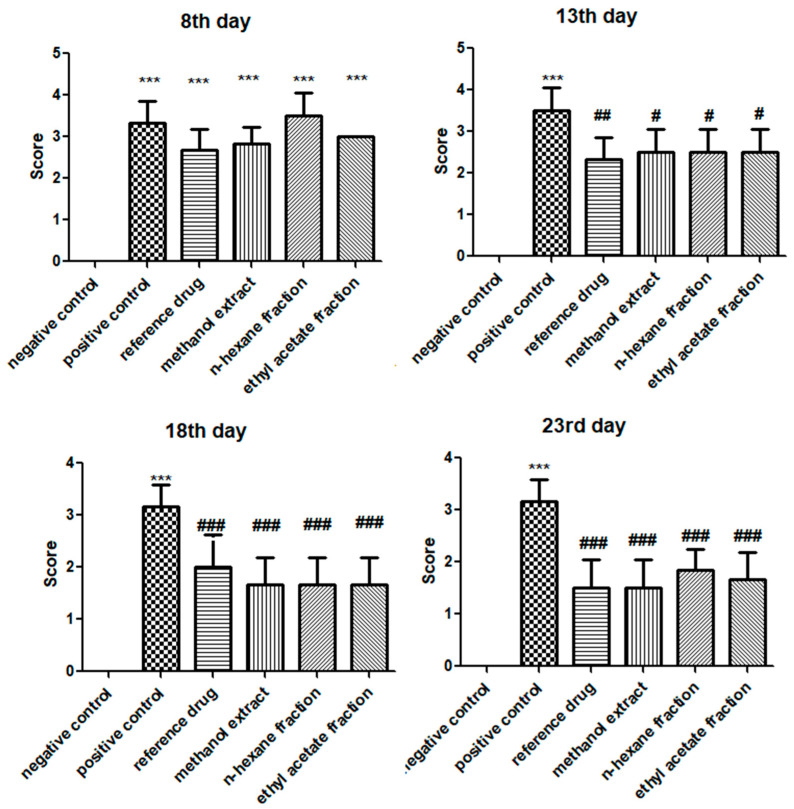
Arthritic score in all groups after arthritic induction using FCA on the 8th, 13th, 18th, and 23rd day. Data are calculated as the mean ± SD (*n* = 6). *** *p* < 0.001 in correlation to the vehicle control group, and # *p* < 0.05, ## *p* < 0.01, and ### *p* < 0.001 in correlation to the arthritic control group.

Arthritic development, manifesting as swelling and erythema, was observed macroscopically after the sub-planter induction of FCA. Treatment with *E. crassipes* was initiated from day 8 until 23. The arthritic control group indicated a significant (*p* < 0.001) escalation in arthritic development (3.33 ± 0.514) compared to that in the vehicle control group since the 8th day. Meanwhile, the plant-treated groups displayed a significant diminution in arthritis in contrast to the positive control on the 13th (methanol extract: 2.5 ± 0.547; *n*-hexane fraction: 2.5 ± 0.547; ethyl acetate fraction: 2.5 ± 0.547), 18th (methanol extract: 1.66 ± 0.516; *n*-hexane fraction: 1.66 ± 0.516; ethyl acetate fraction: 1.66 ± 0.516) and 23rd day (methanol extract: 1.5 ± 0.547; *n*-hexane fraction: 1.833 ± 0.408; ethyl acetate fraction: 1.667 ± 0.516), respectively. On the 18th and 23rd day, the macroscopic observation depicted a significant decrease in arthritis-related swelling and erythema in groups treated with *E. crassipes* compared to that in the positive control group, in which the disease progressed without treatment, as shown in Figure 1. The comparison among all plant-treated groups was statistically non-significant.

### 3.2. E. crassipes Decreased Paw Edema

**Figure 2:**
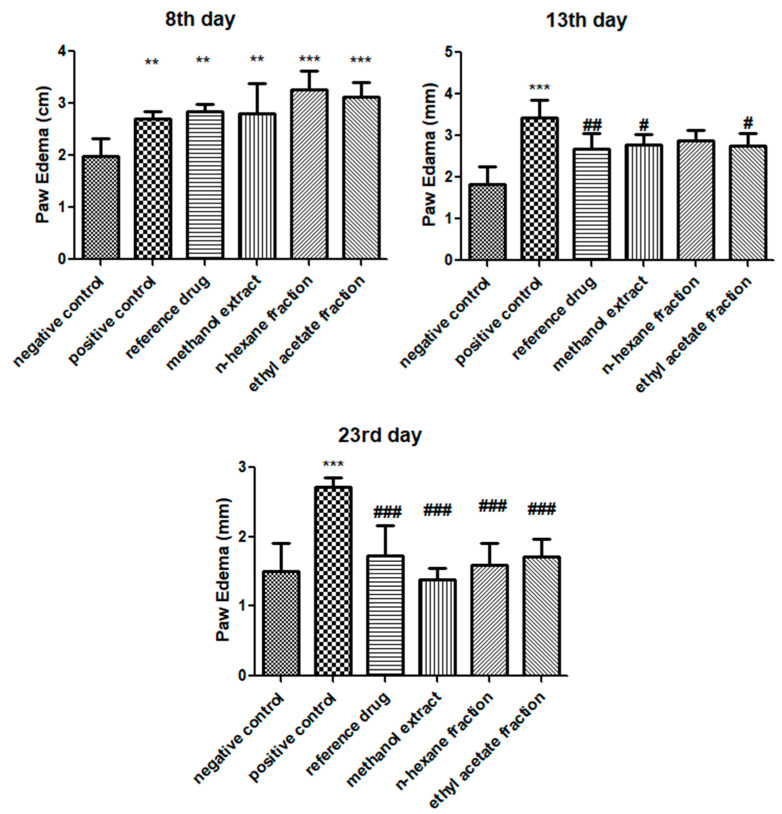
Effect of *E. crassipes* on paw edema. On the 8th day, treatment groups were correlated with the negative control group, while on other days, they were compared with the positive control. Data are calculated as the mean ± SD (*n* = 6). ** *p* < 0.01, *** *p* < 0.001 in correlation to the vehicle control group, and # *p* < 0.05, ## *p* < 0.01, and ### *p*< 0.001 in correlation to the arthritic control group.

After FCA induction, the paw volume was measured in all groups of Sprague–Dawley rats using digital Vernier calipers on the 8th, 13th, and 23rd day. Paw volumes observed on the 8th day exhibited a significant rise in paw edema (arthritic control: 2.71±0.138; methanol extract: 2.812 ± 0.569; *n*-hexane fraction: 3.263 ± 0.362; ethyl acetate fraction: 3.118 ± 0.277) compared to that in the vehicle control group. On the other hand, plant-treated groups displayed a significant decline in paw edema on the 13th day (methanol extract: 2.788 ± 0.25; *n*-hexane fraction: 2.878 ± 0.248; ethyl acetate fraction: 2.748 ± 0.294) compared to that in the positive control group. On the 23rd day, all treated groups (methanol extract: 1.38 ± 0.165; *n*-hexane fraction: 1.587 ± 0.315; ethyl acetate fraction: 1.715 ± 0.247) exhibited significantly reduced levels of paw edema (*p* < 0.001), in contrast to the positive control group, as presented Figure 2. No statistical difference was found among all plant-treated groups.

### 3.3. E. crassipes Attenuated Histopathological Scoring

**Figure 3:**
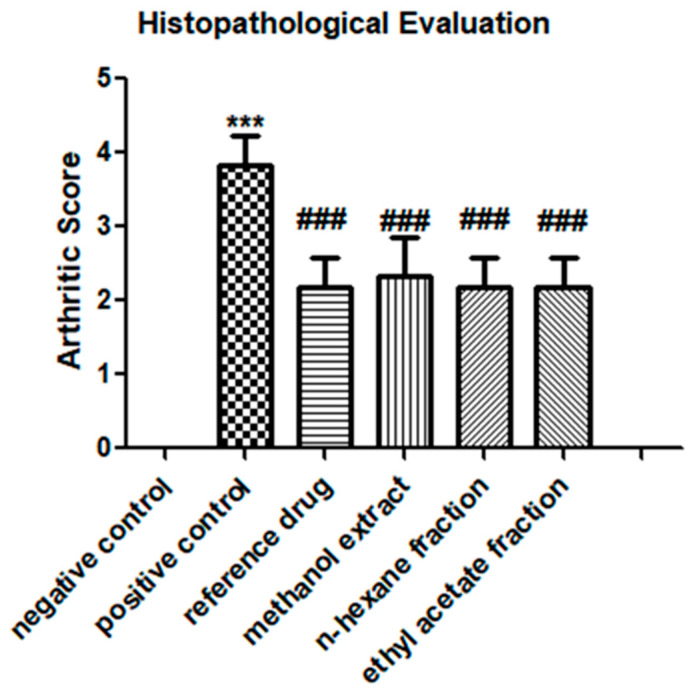
Histopathological evaluation of Sprague–Dawley rats after FCA induction and treatment. Data are calculated as the mean ± SD (*n* = 6). *** *p* < 0.001 in comparison to the vehicle control group and ### *p* < 0.001 in contrast to the arthritic control group.

The histopathological evaluation showed a significant reduction in the arthritic score in the arthritic control group compared to that in the vehicle control group. All treated groups demonstrated an attenuation of the arthritic score at day 23 (methanol extract: 2.333 ± 0.516; *n*-hexane fraction: 2.167 ± 0.408; ethyl acetate fraction: 2.167 ± 0.408) as shown in Figure 3. A comparison between extract- and fraction-treated groups showed non-significant results. 

### 3.4. E. crassipes Down-Regulated TNF-α, IL-1β, IL-6, and NF-κB

**Figure 4:**
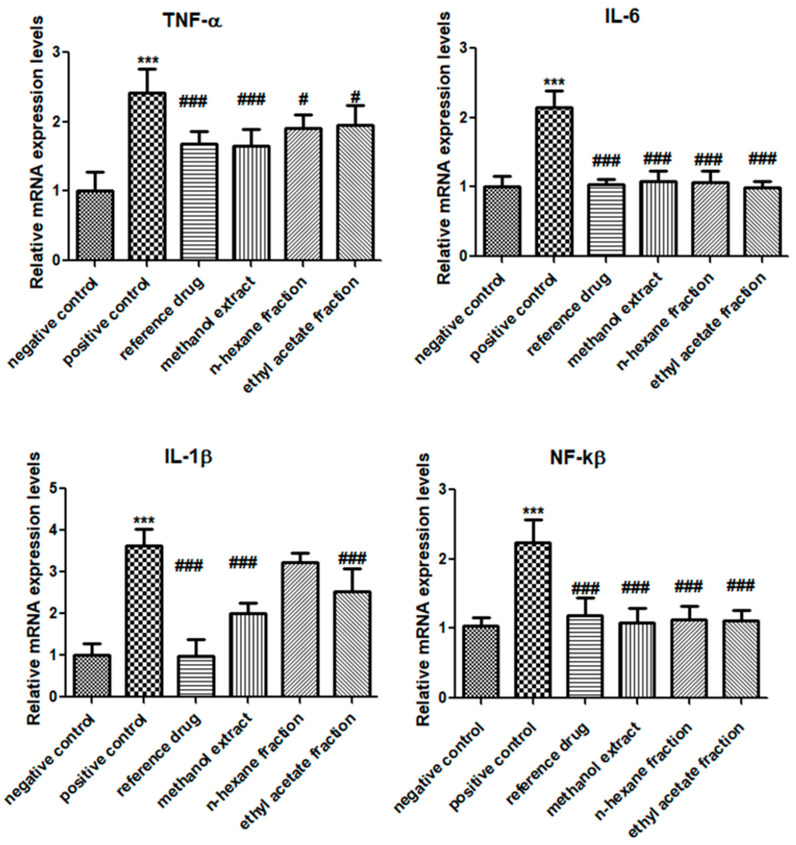
*E. crassipes* reduced the mRNA expression values of TNF-α, IL-1β, IL-6, and NF-κB. Values are stated as the mean ± SD (*n* = 6). *** *p* < 0.001 in contrast to the vehicle control group and # *p* < 0.05 and ### *p* < 0.001 in correlation with the arthritic control group.

Blood samples treated with *E. crassipes* were collected and processed. A significant down-regulation of pro-inflammatory cytokines and matrix metalloproteinase enzymes was found, as shown in Figure 4. The arthritic control showed significant up-regulation of TNF-α (2.413 ± 0.352), IL-1β (3.617 ± 0.409), and IL-6 (2.141 ± 0.242) in comparison to the vehicle control group. In contrast, expression levels of TNF-α were significantly suppressed by the plant (methanol extract: 1.651 ± 0.245; *n*-hexane fraction: 1.911 ± 0.196; ethyl acetate fraction: 1.953 ± 0.277) as compared to those in the arthritic control group. Likewise, expression levels of IL-1β were also significantly reduced in the groups treated with the methanol extract (2.015 ± 0.236) and ethyl acetate fraction (2.517 ± 0.564) as compared to those in the arthritic control group. Similar suppression was shown in the case of the expression levels of IL-6 in the plant-treated groups, as compared to in the positive control (methanol extract: 1.077 ± 0.162; *n*-hexane fraction: 1.063 ± 0.175; ethyl acetate fraction: 0.997 ± 0.085).

The positive control group displayed the significant escalation of NF-κB (2.42 ± 0.319) compared to that in the vehicle control group. Significantly reduced expression of NF-κB was found in the *E. crassipes*-treated groups (methanol extract: 1.087 ± 0.198; *n*-hexane fraction: 1.123 ± 0.204; ethyl acetate fraction: 1.115 ± 0.153) compared to that in the arthritic control group.

### 3.5. E. crassipes Attenuated MMP-2, MMP-3, and VEGF Expression Levels

**Figure 5:**
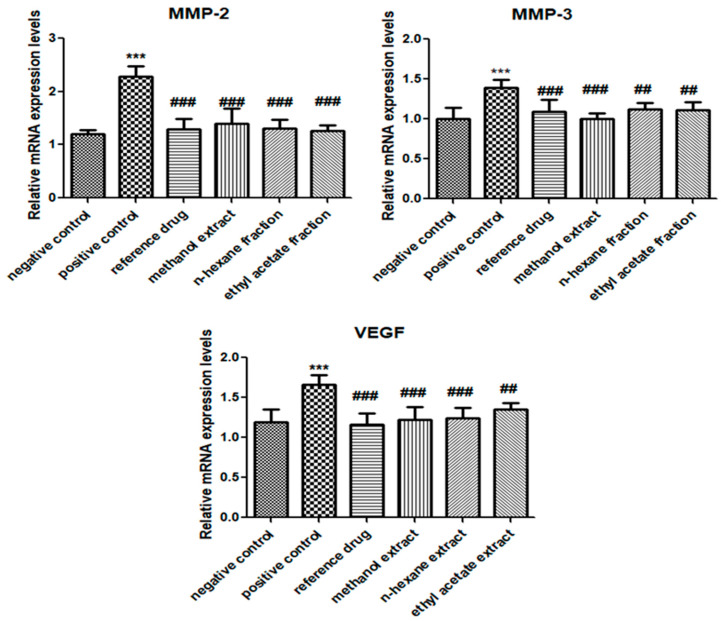
*E. crassipes* reduced the mRNA expression values of MMP-2, MMP-3, and VEGF. Values are calculated as the mean ± SD (*n* = 6). *** *p* < 0.001 in contrast to vehicle control group and ## *p* < 0.001, and ### *p* < 0.001 in contrast to arthritic control group.

The significant up-regulation of MMP-3 (1.393 ± 0.099), MMP-2 (2.279 ± 0.0.194), and VEGF (1.664 ± 0.121) in the arthritic control group was observed as opposed to in the vehicle control group. However, the suppression of MMP-3 levels was found to be statistically significant (methanol extract: 1.002 ± 0.073; *n*-hexane fraction: 1.127 ± 0.076; ethyl acetate fraction: 1.112 ± 0.099) after treatment with *E. crassipes* compared to those in the positive control group. Similarly, expression levels of MMP-2 were also found to be significantly reduced in plant-treated groups (methanol extract: 1.391 ± 0.292; *n*-hexane fraction: 1.307 ± 0.16; ethyl acetate fraction: 1.265 ± 0.105) compared to those in the positive control group. Whereas, similar significant reduction in the expression levels of VEGF was shown after treatment with *E. crassipes* (methanol extract: 1.225 ± 0.159; *n*-hexane fraction: 1.245 ± 0.125; ethyl acetate fraction: 1.349 ± 0.078) compared to those in the arthritic control group. Results are shown in Figure 5.

### 3.6. E. crassipes Significantly Reduced PGE2 Levels

**Figure 6:**
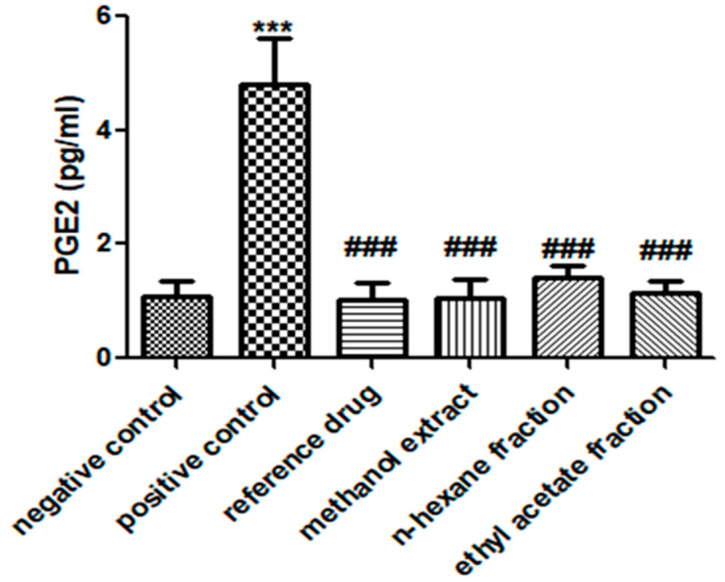
*E. crassipes* significantly declined PGE2 levels in comparison to the arthritic control group. Data are stated as the mean ± SD (*n* = 6). *** *p* < 0.001 in contrast to vehicle control and ### *p* < 0.001 in comparison to arthritic control group.

As indicated in Figure 6, a substantial increase in PGE2 was depicted in the positive control group (4.806 ± 0.796) in comparison to the vehicle control group. However, when groups were treated with *E. crassipes*, they showed a significant decline in PGE2 levels (methanol extract: 1.041 ± 0.355; *n*-hexane fraction: 1.416 ± 0.215; ethyl acetate fraction: 1.134 ± 0.255) compared to those in the arthritic control group, as shown in Figure 6.

### 3.7. E. Crassipes Modulated Hematological Markers

Significant declines (*p* < 0.001) in RBC and the Hb content were detected in the arthritic control group compared to the vehicle control group. In contrast, plant-treated groups showed increases in RBC and the Hb content compared to the positive control group. There was no significant difference found in WBC counts among all groups.

However, the arthritic control group presented with a significant elevation (*p* < 0.001) in the PLT count compared to that in the vehicle control group. Plant-treated groups showed lessened values (*p* < 0.05) of PLT counts compared to those in the arthritic control group. The modulation of hematological markers is shown in Table 2.

### 3.8. E. crassipes Improved Markers of Liver Function Tests

Significantly increased ALT (*p* < 0.001) and AST (*p* < 0.01) values were found in the arthritic control group compared to the vehicle control group. In contrast, in treated groups, hepatic parameters were found to be significantly improved (Table 3). 

### 3.9. No Nephrotoxic Effect of E. crassipes Was Observed

The values of urea and creatinine in all groups were not statistically significant different as compared to each other. This depicts that *E. crassipes* is safe in terms of renal parameters (Table 3).

### 3.10. GC-MS Analysis

The phytochemical composition of the plant was investigated via GC-MS analysis. The compounds found in the plant are known to possess anti-inflammatory and antioxidant properties. The identified compounds are mentioned in the following Table 4.

## 4. Discussion

In our current research, the anti-inflammatory and immunomodulatory properties of *E. crassipes* were evaluated, by using an FCA-induced rat arthritic model. FCA was preferred to induce arthritis in the rat model because the clinical and pathological changes induced by FCA are analogous to those that appear in human RA [28]. A characteristic feature of RA involves the conversion of the synovial membrane into the pannus, with the presence of activated macrophages and synovial fibroblasts at the borderline between the pannus and cartilage [5]. All of this involves the production of inflammatory cytokines, triggered by FCA through toll like receptors [29].

*E. crassipes* is already known for its antioxidant properties [30]. Previously, it had been used for the treatment of swelling, burning, hemorrhage, goiters, irritation, and inflammation [16]. Another member of the Pontederiaceae family i.e., *Monochoria hastata*, also exhibited anti-inflammatory and anti-oxidant effects [31]. Similarly, various plants and phytochemicals belonging to the Cleomaceae family have been shown to possess anti-arthritic and anti-inflammatory properties [32]. A literature review showed that *Embelia ribes* is another herbal medicine that is known to possess anti-oxidant and anti-inflammatory properties and could have anti-arthritic potential [33]. The current study showed that lawsone is the major phytochemical in *E. crassipes*, which is also abundantly found in *Lawsonia inermis* (henna); the latter plant is also known for its analgesic and anti-inflammatory properties [34]. 

The present study showed that *E. crassipes* down-regulated inflammatory markers, both in vitro and in vivo, and attenuated the development of RA. The histopathological examination of diseased groups depicted the increased penetration of inflammatory cells into the synovium, resulting in bone erosion and pannus formation. The data showed the amelioration of histopathological parameters in the plant-treated groups. 

The effects of *E. crassipes* were on the levels of pro-inflammatory cytokines (TNF-α, IL-1β, IL-6, NF-ĸB, and VEGF) and matrix metalloproteinase enzymes (MMP-2 and MMP-3) [35]. An increased concentration of TNF-α results in inflammation and articular damage [36]. IL-1β is known as a cytokine responsible for the bone erosion and pannus formation in RA [37]. NF-ĸB triggers the proliferation and inhibits the apoptosis of fibroblast-like synoviocytes in the synovial intimal lining, resulting in the destruction of cartilage and bone damage [38]. The stress-induced signaling of NF-ĸB triggers the formation of IL-6, through Angiotensin II, cytokines, ROS stress, and vascular injury, which is the most significant signaling factor in regulating inflammation, synovial hyperplasia, and matrix degeneration. This may further lead to osteoporosis and bone destruction [37]. Likewise, VEGF is notorious for producing synovial inflammation, hyperplasia, and angiogenesis in the RA-affected joints [39]. Treatment with the plant methanol extract and its fractions attenuated the mRNA expression levels of inflammatory markers, which are mostly known to be amplified in immunomodulatory diseases [40,41,42,43]. This down-regulation of these pro-inflammatory markers, mediated by the plant extract, indicated that the amelioration of RA could be ascribed to the plant’s potential immunomodulatory and anti-inflammatory properties [44].

PGE2 is another important marker that plays a significant inflammatory role in RA [45]. It is responsible for the bone erosion, the destruction of articular cartilage, vasodilation, the extravasation of fluid, and acute pain in RA [46]. *E. crassipes* also attenuated the PGE2 levels during the progression of RA.

In the current study, our results depicted the reduced values of RBC counts and Hb contents in the positive control group, which indicates the anemic condition of rats. Anemia may be explained due to deficiencies in the generation of cells as a result of a decline in bone marrow functioning and/or reduced iron storage in the reticuloendothelial system [47]. In contrast, a rise in PLT counts in the positive control group indicates the stimulation of an immunomodulatory reaction due to the attacking pathogen [48]. Treatment with *E. crassipes* almost stabilized the levels of evaluated hematological parameters (WBC, RBC, PLT, and Hb content). Likewise, an evaluation of hepatic and renal markers depicted the safety of *E. crassipes* in terms of hepatotoxicity or nephrotoxicity.

GC-MS analysis also indicated the phytochemical components of the plant that are considered to possess anti-oxidant and anti-inflammatory properties [49]. Lawsone was found in the highest concentration in the methanol extract of *E. crassipes*. Previous studies have documented the anti-inflammatory activity of lawsone in different in vivo inflammatory models, like pancreatitis and carrageenan-induced inflammatory edema, and are suggestive of its effect through the modulation of TNFα and NF-кB [50,51]. Different fatty acids are also found in high concentrations. Octadecanoic acid possesses strong binding affinity for MMP2, as shown via a molecular docking analysis, and thus contributes to the anti-inflammatory effect [52]. Hexadecanoic acid inhibits phospholipase A2 and therefore may be considered anti-inflammatory [53]. Malonic acid was previously reported to attenuate the expression of MMPs, anti-oxidant enzymes, and pro-inflammatory cytokines [54]. A recent study suggested that stigmasterol possesses an anti-inflammatory property, which might be ascribed to glucocorticoid receptors [55]. Another study showed the protective effect of malonic acid against cartilage degradation, partially through NF-кB inhibition [55]. Squalene is also known to possess anti-oxidant and anti-inflammatory activities, which might be attributed to the down-regulation of COX2 and TNFα levels [56]. It is plausible that the anti-inflammatory effect of *E. crassipes*, determined in this study, is due to the presence of these phytochemical constituents. Further studies are required to determine the anti-arthritic effect of isolated phytochemicals. Future studies may also consider the possibility of incorporating these phytochemicals in nanoscale-drug-delivery systems, which could increase the bioavailability and solubility of phytochemicals, leading to reduced dosages [57].

## 5. Conclusions

The data showed that *E. crassipes* possesses significant anti-inflammatory and anti-arthritic properties against FCA-induced RA in an animal model. Treatment with *E. crassipes* reduced arthritic progress and paw edema along with histopathological parameters. The diminution of RA may be attributed to the decline in inflammatory parameters, like TNF-α, IL-1β, IL-6, NF-ĸB, MMP-2, MMP-3, and VEGF, mediated by the plant. *E. crassipes* also reduced the levels of PGE2. Results of *E. crassipes* flowers were also found to be comparable to piroxicam in the reduction of RA. The statistical analysis showed no difference in the anti-arthritic effect of the methanol extract and fractions on the 23rd day. The study used GC-MS analysis for the identification of compounds; however, further LC-MS analysis is recommended to identify a broader range of compounds. Future studies may consider evaluating the phytochemical constituents present in *E. crassipes* for their possible anti-arthritic activity. The effects of *E. crassipes* on different signaling pathways may also be explored. Clinical data may also be generated in future studies for an exploration of the possible use of *E. crassipes* as an adjunct therapy with conventional medicines.

## Figures and Tables

**Table 1:** Primers.

Genes	Forward Primer	Reverse Primer	Annealing Temperature	Product Size	
IL-1β	5′-CCTGCTAGTGTGTGATGTTC-3′	5′-GAGGTGCTGATGTACCAGTT-3′	58 °C	390	ENSRNOG0000004649
IL-6	5′-AGACTTCCAGCCAGTTGCCT-3′	5′-CTGACAGTGCATCATCGCTG-3′	60 °C	233	[24]
TNF-α	5′-CCTCTTCTCATTCCTGCTCGT-3″	5′-TGAGATCCATGCCATTGGCC-3′	60 °C	266	[25]
NF-κB	5′-CAAGGAAGAGGATGTGGGGTT-3	5′-AGCTGAGCATGAAGGTGGATG-3′	60 °C	207	[25]
VEGF	5′-GTTCAGAGCGGAGAAAGCATT-3	5′-CTTGCAACGCGAGTCTGTGT-3′	60 °C	80	[26]
MMP-2	5′-CGAACAAGTATGAGAGCTGC-3′	5′-CGGTCATCATCGTAGTTGGT-3′	57 °C	85	ENSRNOG00000016695
MMP-3	5′-CCTTTTGATGGGCCTGGAAT-3′	5′-GTGACATCATCTGTCCATCG-3′	58 °C	107	ENSRNOG00000032626
GAPDH	5′-GTCATCAACGGGAAACCCAT-3′	5′-CTTGCCGTGGGTAGAGTCAT-3	60 °C	229	[27]

**Table 2:** *E. crassipes* modulated hematological markers: Data are stated as the mean ± SD (*n* = 6). ** *p* < 0.01 and *** *p* < 0.001 in contrast to the negative control group and # *p* < 0.05, ## *p* < 0.01, and ### *p* < 0.001 in correlation with the positive control group.

Hematological Parameters	Vehicle Control Group	Arthritic Control Group	Reference Drug Group	Methanol Extract	*n*-hexane Fraction	Ethyl Acetate Fraction
RBC	8.29 ± 0.666	6.9 ± 0.6 ***	7.9 ± 0.3 ##	7.66 ± 0.275 #	7.81 ± 0.32 #	7.64 ± 0.46 #
WBC	9.60 ± 1.607	10.75 ± 0.736	9.7 ± 1.288	10.15 ± 0.717	9.47 ± 1.3	9.68 ± 1.422
PLT	660.5 ± 37.05	776.7 ± 48.12 **	684.2 ± 62.86 #	691.5 ± 42.93 #	685.3 ± 25.46 #	685.2 ± 42.44 #
Hb	11.73 ± 0.326	10.35 ± 0.796 ***	11.80 ± 0.334 ###	11.88 ± 0.354 ###	11.75 ± 0.301 ###	11.78 ± 0.23 ###

**Table 3:** *E. crassipes* improved markers of liver function tests and showed no nephrotoxic effect: values are stated as the mean ± SD (*n* = 6). ** *p* < 0.01 and *** *p* < 0.001 in contrast to the vehicle control and # *p* < 0.05 and ## *p* < 0.01 in contrast to arthritic control group.

Biochemical Parameters	Vehicle Control Group	Arthritic Control Group	Reference Drug Group	Methanol Extract	*n*-hexane Fraction	Ethyl Acetate Fraction
ALT	41.33 ± 9.180	59.50 ± 3.391 ***	47.83 ± 4.792 #	48.83 ± 6.21 #	45.67 ± 5.046 ##	44.67 ± 4.590 ##
AST	278.3 ± 58.29	386.7 ± 44.57 **	291.0 ± 27.21 ##	298.5 ± 41.23 ##	307.2 ± 13.0 #	289.7 ± 46.74 ##
Urea	19.83 ± 5.981	19.5 ± 2.665	17.17 ± 2.48	16.33 ± 3.83	15.83 ± 3.371	15.83 ± 5.345
Creatinine	0.666 ± 0.081	0.65 ± 0.054	0.616 ± 0.04	0.65 ± 0.083	0.633 ± 0.081	0.6 ± 0.089

**Table 4:** Phytochemical components detected through mass chromatograms of the methanol extract of *E. crassipes*.

Sr. No.	Retention Time (Min)	Area %	Name of Detected Compound	Class of Phytochemical Compound	Molecular Formula	Molecular Weight (g/mol)	Structure
1	8.691	14.62	2-Hydroxy-1,4-naphthoquinone (Lawsone)	Naphthoquinone	C_10_H_6_O_3_	174.15	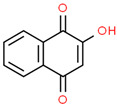
2	17.200	12.39	Octadecanoic acid	Fatty Acid	C_18_H_36_O_2_	284.48	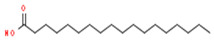
3	11.375	11.375	Pentadecanoic acid	Fatty Acid	C_15_H_30_O_2_	242.39	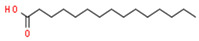
4	17.201	3.22	Hexadecanoic acid	Fatty Acid	C_16_H_32_O_2_	256.4	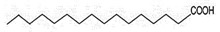
5	13.328	0.90	Linoleic acid	Omega-6 Fatty Acid	C18H32O2	280.44	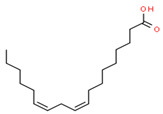
6	23.4	0.85	Vaccenic acid	Fatty Acid	C_18_H_34_O_2_	282.461	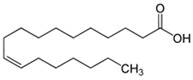
7	15.46	0.80	Malonic acid	Dicarboxylic acid	C_3_H_4_O_4_	104.0615	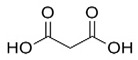
8	31.51	0.78	β-stigmasterol	Stigmastane	C_29_H_50_O	414.71	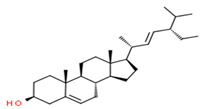
9	14.83	0.75	Caprylic acid	Fatty Acid	C_8_H_16_O_2_	144.21	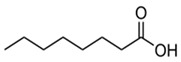
10	16.12	0.70	Nonanoic acid	Fatty Acid	C_9_H_18_O_2_	158.23	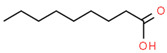
11	18.92	0.65	Myristic acid	Fatty Acid	C14H28O2	228.37	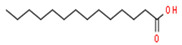
12	28.5	0.50	Squalene	Triterpenoid	C30H50	410.73	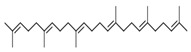

## Data Availability

Data will be provided on demand.

## References

[1] Sun X.-B., Liu Y.-P., Yang Y.-Y., Liu X.-Y., Xiang D.-X. (2016). Anti-arthritic effect of total saponins from Clematis henryi Oliv. on collagen-induced arthritis rats. Eur. J. Inflamm..

[2] Monti S., Montecucco C., Bugatti S., Caporali R. (2015). Rheumatoid arthritis treatment: The earlier the better to prevent joint damage. RMD Open.

[3] Romao V.C., Fonseca J.E. (2021). Etiology and Risk Factors for Rheumatoid Arthritis: A State-of-the-Art Review. Front. Med..

[4] Sudoł-Szopińska I., Kontny E., Maśliński W., Prochorec-Sobieszek M., Kwiatkowska B., Zaniewicz-Kaniewska K., Warczyńska A. (2012). The pathogenesis of rheumatoid arthritis in radiological studies. Part I: Formation of inflammatory infiltrates within the synovial membrane. J. Ultrason..

[5] Gautam R.K., Singh D., Nainwani R. (2013). Medicinal plants having anti-arthritic potential: A review. Int. J. Pharm. Sci. Rev. Res.

[6] Radu A.F., Bungau S.G., Negru P.A., Marcu M.F., Andronie-Cioara F.L. (2022). In-depth bibliometric analysis and current scientific mapping research in the context of rheumatoid arthritis pharmacotherapy. Biomed. Pharmacother. Biomed. Pharmacother..

[7] Avau A., Mitera T., Put S., Put K., Brisse E., Filtjens J., Uyttenhove C., Van Snick J., Liston A., Leclercq G. (2014). Systemic juvenile idiopathic arthritis–like syndrome in mice following stimulation of the immune system with Freund’s complete adjuvant: Regulation by interferon-γ. Arthritis Rheumatol..

[8] Cross M., Smith E., Hoy D., Carmona L., Wolfe F., Vos T., Williams B., Gabriel S., Lassere M., Johns N. (2014). The global burden of rheumatoid arthritis: Estimates from the global burden of disease 2010 study. Ann. Rheum. Dis..

[9] Machado-Duque M.E., Ramirez-Valencia D.M., Murillo-Munoz M.M., Machado-Alba J.E. (2020). Trends in Opioid Use in a Cohort of Patients with Rheumatoid Arthritis. Pain Res. Manag..

[10] Yap H.Y., Tee S.Z., Wong M.M., Chow S.K., Peh S.C., Teow S.Y. (2018). Pathogenic Role of Immune Cells in Rheumatoid Arthritis: Implications in Clinical Treatment and Biomarker Development. Cells.

[11] Zago B.A., Priyadharshini A., Vijayakumar T.M. (2020). Safety and efficacy of newer biologics DMARDs in the management of rheumatoid arthritis: A systematic review. Osteoarthr. Cartil. Open.

[12] Dixon W.G., Hyrich K.L., Watson K.D., Lunt M., Galloway J., Ustianowski A., Consortium B.S.R.B.R.C.C., Symmons D.P., Register B.S.R.B. (2010). Drug-specific risk of tuberculosis in patients with rheumatoid arthritis treated with anti-TNF therapy: Results from the British Society for Rheumatology Biologics Register (BSRBR). Ann. Rheum. Dis..

[13] Park D.W., Kim Y.J., Sung Y.K., Chung S.J., Yeo Y., Park T.S., Lee H., Moon J.Y., Kim S.H., Kim T.H. (2022). TNF inhibitors increase the risk of nontuberculous mycobacteria in patients with seropositive rheumatoid arthritis in a mycobacterium tuberculosis endemic area. Sci. Rep..

[14] Edwards C.J. (2005). Immunological therapies for rheumatoid arthritis. Br. Med. Bull..

[15] Gichuki J., Omondi R., Boera P., Okorut T., Matano A.S., Jembe T., Ofulla A. (2012). Water hyacinth *Eichhornia crassipes* (Mart.) Solms-Laubach dynamics and succession in the Nyanza Gulf of Lake Victoria (east Africa): Implications for water quality and biodiversity conservation. Sci. World J..

[16] Jayanthi P., Lalitha P., Sujitha R., Thamaraiselvi A. (2013). Anti-inflammatory activity of the various solvent extracts of *Eichhornia crassipes* (Mart.) Solms. Int. J. Pharm. Tech. Res..

[17] Fileto-Pérez H.A., Rutiaga-Quiñones O.M., Sytsma M.D., Lorne I.M., Luo W., Pankow J.F., Rutiaga-Quiñones J.G. (2015). GC/MS analysis of some extractives from *Eichhornia crassipes*. BioResources.

[18] Ijaz B., Shabbir A., Shahzad M., Mobashar A., Sharif M., Basheer M.I., Tareen R.B., Syed N.I. (2021). Amelioration of airway inflammation and pulmonary edema by Teucrium stocksianum via attenuation of pro-inflammatory cytokines and up-regulation of AQP1 and AQP5. Respir. Physiol. Neurobiol..

[19] Shabbir A., Batool S.A., Basheer M.I., Shahzad M., Sultana K., Tareen R.B., Iqbal J. (2018). Ziziphora clinopodioides ameliorated rheumatoid arthritis and inflammatory paw edema in different models of acute and chronic inflammation. Biomed. Pharmacother..

[20] Kyei S., Koffuor G.A., Boampong J.N. (2012). Antiarthritic effect of aqueous and ethanolic leaf extracts of *Pistia stratiotes* in adjuvant-induced arthritis in Sprague-Dawley rats. J. Exp. Pharmacol..

[21] Akhtar G., Shabbir A. (2019). Urginea indica attenuated rheumatoid arthritis and inflammatory paw edema in diverse animal models of acute and chronic inflammation. J. Ethnopharmacol..

[22] Mobashar A., Shabbir A., Shahzad M., Gobe G. (2022). Preclinical rodent models of arthritis and acute inflammation indicate Immunomodulatory and Anti-Inflammatory Properties of Juglans regia Extracts. Evid. Based Complement. Altern. Med..

[23] Cai X., Zhou H., Wong Y.F., Xie Y., Liu Z.Q., Jiang Z.H., Bian Z.X., Xu H.X., Liu L. (2007). Suppression of the onset and progression of collagen-induced arthritis in rats by QFGJS, a preparation from an anti-arthritic Chinese herbal formula. J. Ethnopharmacol..

[24] Uttra A.M., Alamgeer, Shahzad M., Shabbir A., Jahan S. (2018). Ephedra gerardiana aqueous ethanolic extract and fractions attenuate Freund Complete Adjuvant induced arthritis in Sprague Dawley rats by downregulating PGE2, COX2, IL-1β, IL-6, TNF-α, NF-kB and upregulating IL-4 and IL-10. J. Ethnopharmacol..

[25] Shabbir A., Shahzad M., Ali A., Zia-Ur-Rehman M. (2016). Discovery of New Benzothiazine Derivative as Modulator of Pro- and Anti-inflammatory Cytokines in Rheumatoid Arthritis. Inflammation.

[26] Zhu J., Su C., Chen Y., Hao X., Jiang J. (2019). Electroacupuncture on ST36 and GB39 Acupoints Inhibits Synovial Angiogenesis via Downregulating HIF-1α/VEGF Expression in a Rat Model of Adjuvant Arthritis. Evid. Based Complement. Altern. Med..

[27] Shabbir A., Shahzad M., Ali A., Zia-ur-Rehman M. (2014). Anti-arthritic activity of N′-[(2,4-dihydroxyphenyl)methylidene]-2-(3,4-dimethyl-5,5-dioxidopyrazolo[4,3-c][1,2]benzothiazin-1(4*H*)-yl)acetohydrazide. Eur. J. Pharmacol..

[28] Nasuti C., Fedeli D., Bordoni L., Piangerelli M., Servili M., Selvaggini R., Gabbianelli R. (2019). Anti-Inflammatory, Anti-Arthritic and Anti-Nociceptive Activities of Nigella sativa Oil in a Rat Model of Arthritis. Antioxidants.

[29] Gupta S., Mishra K.P., Kumar B., Singh S.B., Ganju L. (2020). Andrographolide attenuates complete freund’s adjuvant induced arthritis via suppression of inflammatory mediators and pro-inflammatory cytokines. J. Ethnopharmacol..

[30] Aboul-Enein A.M., Al-Abd A.M., Shalaby E., Abul-Ela F., Nasr-Allah A.A., Mahmoud A.M., El-Shemy H.A. (2011). *Eichhornia crassipes* (Mart) solms: From water parasite to potential medicinal remedy. Plant Signal Behav..

[31] Haq M.M., Chowdhury M.A.R., Tayara H., Abdelbaky I., Islam M.S., Chong K.T., Jeong S. (2021). A Report on Multi-Target Anti-Inflammatory Properties of Phytoconstituents from *Monochoria hastata* (Family: Pontederiaceae). Molecules.

[32] Khuntia A., Martorell M., Ilango K., Bungau S.G., Radu A.F., Behl T., Sharifi-Rad J. (2022). Theoretical evaluation of *Cleome* species’ bioactive compounds and therapeutic potential: A literature review. Biomed. Pharmacother. Biomed. Pharmacother..

[33] Sharma V., Gautam D.N.S., Radu A.F., Behl T., Bungau S.G., Vesa C.M. (2022). Reviewing the Traditional/Modern Uses, Phytochemistry, Essential Oils/Extracts and Pharmacology of *Embelia ribes* Burm. Antioxidants.

[34] Yogisha S., Samiulla D.S., Prashanth D., Padmaja R., Amit A. (2002). Trypsin inhibitory activity of Lawsonia inermis. Fitoterapia.

[35] Araki Y., Mimura T. (2017). Matrix Metalloproteinase Gene Activation Resulting from Disordred Epigenetic Mechanisms in Rheumatoid Arthritis. Int. J. Mol. Sci..

[36] Moelants E.A., Mortier A., Van Damme J., Proost P. (2013). Regulation of TNF-alpha with a focus on rheumatoid arthritis. Immunol. Cell Biol..

[37] Chen J., Wu W., Zhang M., Chen C. (2019). Taraxasterol suppresses inflammation in IL-1beta-induced rheumatoid arthritis fibroblast-like synoviocytes and rheumatoid arthritis progression in mice. Int. Immunopharmacol..

[38] Ding Q., Hu W., Wang R., Yang Q., Zhu M., Li M., Cai J., Rose P., Mao J., Zhu Y.Z. (2023). Signaling pathways in rheumatoid arthritis: Implications for targeted therapy. Signal Transduct. Target. Ther..

[39] Yoo S.A., Kwok S.K., Kim W.U. (2008). Proinflammatory role of vascular endothelial growth factor in the pathogenesis of rheumatoid arthritis: Prospects for therapeutic intervention. Mediat. Inflamm..

[40] Seibl R., Birchler T., Loeliger S., Hossle J.P., Gay R.E., Saurenmann T., Michel B.A., Seger R.A., Gay S., Lauener R.P. (2003). Expression and regulation of Toll-like receptor 2 in rheumatoid arthritis synovium. Am. J. Pathol..

[41] Huang Q., Ma Y., Adebayo A., Pope R.M. (2007). Increased macrophage activation mediated through toll-like receptors in rheumatoid arthritis. Arthritis Rheum. Off. J. Am. Coll. Rheumatol..

[42] Kim H.-R., Cho M.-L., Kim K.-W., Juhn J.-Y., Hwang S.-Y., Yoon C.-H., Park S.-H., Lee S.-H., Kim H.-Y. (2007). Up-regulation of IL-23p19 expression in rheumatoid arthritis synovial fibroblasts by IL-17 through PI3-kinase-, NF-κB-and p38 MAPK-dependent signalling pathways. Rheumatology.

[43] Gheorghe K.R., Korotkova M., Catrina A.I., Backman L., Af Klint E., Claesson H.-E., Rådmark O., Jakobsson P.-J. (2009). Expression of 5-lipoxygenase and 15-lipoxygenase in rheumatoid arthritis synovium and effects of intraarticular glucocorticoids. Arthritis Res. Ther..

[44] Noufal K.P., Rajesh B., Nair S.S. (2022). Antiproliferative Effects of the Methanolic Petiole Extract of *Eichhornia crassipes* Against Sloan Kettering Melanoma 5 Cell Line: An In Vitro Study. Cureus.

[45] Jia X.Y., Chang Y., Sun X.J., Dai X., Wei W. (2014). The role of prostaglandin E2 receptor signaling of dendritic cells in rheumatoid arthritis. Int. Immunopharmacol..

[46] Fattahi M.J., Mirshafiey A. (2012). Prostaglandins and rheumatoid arthritis. Arthritis.

[47] Hashimoto M., Fujii T., Hamaguchi M., Furu M., Ito H., Terao C., Yamamoto K., Yamamoto W., Matsuo T., Mori M. (2014). Increase of hemoglobin levels by anti-IL-6 receptor antibody (tocilizumab) in rheumatoid arthritis. PLoS ONE.

[48] Ekambaram S., Perumal S.S., Subramanian V. (2010). Evaluation of antiarthritic activity of *Strychnos potatorum* Linn seeds in Freund’s adjuvant induced arthritic rat model. BMC Complement. Altern. Med..

[49] Shanab S.M., Shalaby E.A., Lightfoot D.A., El-Shemy H.A. (2010). Allelopathic effects of water hyacinth [*Eichhornia crassipes*]. PLoS ONE.

[50] Vanco J., Travnicek Z., Hosek J., Suchy P. (2017). In vitro and in vivo anti-inflammatory active copper(II)-lawsone complexes. PLoS ONE.

[51] Sandeep Birdar B.V. (2012). Protective effects of Lawsone in L-Arginine induced pancreatitis in rats. Indian J. Exp. Biol..

[52] Manivannan P., Muralitharan G., Balaji N.P. (2017). Prediction aided in vitro analysis of octa-decanoic acid from Cyanobacterium *Lyngbya* sp. as a proapoptotic factor in eliciting anti-inflammatory properties. Bioinformation.

[53] Aparna V., Dileep K.V., Mandal P.K., Karthe P., Sadasivan C., Haridas M. (2012). Anti-inflammatory property of n-hexadecanoic acid: Structural evidence and kinetic assessment. Chem. Biol. Drug Des..

[54] Park C., Park J., Kim W.J., Kim W., Cheong H., Kim S.J. (2021). Malonic Acid Isolated from *Pinus densiflora* Inhibits UVB-Induced Oxidative Stress and Inflammation in HaCaT Keratinocytes. Polymers.

[55] Gabay O., Sanchez C., Salvat C., Chevy F., Breton M., Nourissat G., Wolf C., Jacques C., Berenbaum F. (2010). Stigmasterol: A phytosterol with potential anti-osteoarthritic properties. Osteoarthr. Cartil..

[56] Zhang P., Liu N., Xue M., Zhang M., Xiao Z., Xu C., Fan Y., Liu W., Qiu J., Zhang Q. (2023). Anti-Inflammatory and Antioxidant Properties of Squalene in Copper Sulfate-Induced Inflammation in Zebrafish (*Danio rerio*). Int. J. Mol. Sci..

[57] Radu A.F., Bungau S.G. (2023). Nanomedical approaches in the realm of rheumatoid arthritis. Ageing Res. Rev..

